# Optimizing ensemble U-Net architectures for robust coronary vessel segmentation in angiographic images

**DOI:** 10.1038/s41598-024-57198-5

**Published:** 2024-03-19

**Authors:** Shih-Sheng Chang, Ching-Ting Lin, Wei-Chun Wang, Kai-Cheng Hsu, Ya-Lun Wu, Chia-Hao Liu, Yang C. Fann

**Affiliations:** 1 Division of Cardiovascular Medicine, China Medical University Hospital, Taichung, Taiwan; 2 Artificial Intelligence Center, China Medical University Hospital, Taichung, Taiwan; 3 Department of Neurology, China Medical University Hospital, Taichung, Taiwan; 4 Neuroscience and Brain Disease Center, China Medical University, Taichung, Taiwan; 5 School of Medicine, College of Medicine, China Medical University, Taichung, Taiwan; 6 Division of Intramural Research, National Institute of Neurological Disorders and Stroke, National Institutes of Health, 35 Convent Dr., Bethesda, MD 20892 USA; 7 Artificial Intelligence Center, China Medical University Hospital, Taichung, Taiwan

**Keywords:** Interventional cardiology, Machine learning

## Abstract

Automated coronary angiography assessment requires precise vessel segmentation, a task complicated by uneven contrast filling and background noise. Our research introduces an ensemble U-Net model, SE-RegUNet, designed to accurately segment coronary vessels using 100 labeled angiographies from angiographic images. SE-RegUNet incorporates RegNet encoders and squeeze-and-excitation blocks to enhance feature extraction. A dual-phase image preprocessing strategy further improves the model's performance, employing unsharp masking and contrast-limited adaptive histogram equalization. Following fivefold cross-validation and Ranger21 optimization, the SE-RegUNet 4GF model emerged as the most effective, evidenced by performance metrics such as a Dice score of 0.72 and an accuracy of 0.97. Its potential for real-world application is highlighted by its ability to process images at 41.6 frames per second. External validation on the DCA1 dataset demonstrated the model's consistent robustness, achieving a Dice score of 0.76 and an accuracy of 0.97. The SE-RegUNet 4GF model's precision in segmenting blood vessels in coronary angiographies showcases its remarkable efficiency and accuracy. However, further development and clinical testing are necessary before it can be routinely implemented in medical practice.

## Introduction

Coronary Artery Disease (CAD), the leading cause of cardiovascular death worldwide, is responsible for almost 7 million fatalities annually^1^. Coronary angiography remains the gold standard for diagnosing the presence and severity of coronary artery diseases, guiding revascularization strategies. The findings from coronary angiography also have prognostic implications for long-term outcomes^2^. Patients eligible for invasive coronary angiography, presenting with significant stenosis (> 70%) in a major vessel or stenosis > 50% alongside a fractional flow reserve (FFR) below 0.8, should undergo evaluation for revascularization therapy. In deciding between percutaneous coronary intervention (PCI) and coronary artery bypass graft (CABG), a thorough evaluation of vascular lesion severity and associated surgical risks is essential^3^.

However, accurately interpreting coronary angiography requires extensive training and can be subjective due to challenges like multiple viewing angles, dynamic images, overlapping structures, and uneven contrast enhancement. These factors contribute to inconsistency and inefficiency in current medical practices^4^. Earlier studies with semi-automated methods often required extensive manual corrections from cardiologists or trained experts^5^, which makes it not widely adopted in routine clinical use. Additionally, the common practice among hospitals of documenting important vascular lesions through textual summaries would further intensify the difficulties of analyzing the angiographic characteristics^6^. As a result, if detailed information about coronary artery lesions is needed for analysis, it often means revisiting and reexamining the angiograms to locate the necessary information. Therefore, fast and better interpretation approaches are needed to objectively extract detailed anatomy and pathology from complex angiographic data to assist with the clinical assessment of CAD patients.

Automatic segmentation of coronary vessels is often considered the starting point for automated angiography analysis. Extracting vasculature from coronary angiography can be a complex process, which makes obtaining accurate vessel anatomy critical for detecting abnormal segments and measuring stenosis. While recent advances in employing machine learning algorithms have shown promise in assisting diagnosis for various medical imaging modalities^7^, developing consistent and accurate algorithms for coronary angiography interpretation remains challenging^8,9^. Utilizing deep learning models such as U-Net for medical image segmentation has shown great potential; however, applying these techniques to coronary angiography poses unique challenges, especially in real-world clinical applications, Table 1. Recent deep learning architectures still show insufficient performance in images with small vessels, severe vascular stenosis, or poor image quality^10–14^. Occasionally, the preprocessing of images may result in the loss of detail or distortion^15^. Moreover, the lack of uniform evaluation criteria across studies hinders direct comparison of performance metrics. Variability also exists in the definition of areas of interest within vessel masks; while the majority of studies encompass the three coronary artery trees, others concentrate exclusively on the major coronary arteries, or evaluate a single coronary artery in isolation^10,16^. Such discrepancies add to the challenge of comparing results across different studies.

**Table 1:** Comparative analysis of coronary artery segmentation algorithms: performance, limitations, and dataset size.

References	No. of angiograms	Algorithm	Results	Limits
Cervantes-Sanchez et al.^30^	130	Multiscale ANN	ACC: 0.97 DICE: 0.69	High computational demand; difficulties near major vessels
Yang et al.^10^	3302	U-Net with Advanced CNN Encoders	F1: 0.94	Limited to single and major coronary arteries; issues with LCA and stenotic regions
Li et al.^11^	538	CAU-net	ACC: 0.99 DICE: 0.90	Requires DSA images; suboptimal performance on small vessels
Shi et al.^37^	4000	UENet: U-Net generator with multi-scale discriminator	MPA: 0.84	Requires binary images for input
Zhou et al.^16^	102	U-Net	F1: 0.89	Focuses only on RCA and main coronary arteries; problematic at bifurcations
Iyer et al.^12^	462	AngioNet: Deeplab v3+ with APN	ACC: 0.98 DICE: 0.86	Tends to overestimate vessel boundaries in severe stenosis; issues with sharp diameter changes
Algarni et al.^13^	130	Attention-based nested U-net	ACC: 0.97 DICE: 0.92	Difficulties with small vessels and lower-quality images
Menezes et al.^14,34^	416	EfficientUNet ++	ACC: 0.99 DICE: 0.95	Struggles with catheter discrimination, poor image quality, and severe stenosis
Roy et al.^35^	28	U-Net	ACC: 0.98	Limited by a small dataset; concerns over broad applicability
Meng et al.^17^	616	U-Net 3+	DICE: 0.89	
Shen et al.^36^	70	DBCU-Net: U-Net combining DenseNet and bi-directional ConvLSTM	ACC: 0.99 F1: 0.88	Small dataset size; questions regarding generalizability
Fu et al.^15^	217	TV-TRPCA, TSRG	F1: 0.93	Filtering process may reduce precision
Zhang et al.^38^	1000	CIDN: U-Net, introducing BAB and MIB	ACC: 0.98 F1: 0.87	

*ACC* accuracy, *DICE* dice coefficient, *MPA* mean pixel accuracy, *F1* F1 score, *TV-TRPCA* total variation-tensor robust principal component analysis, *TSRG* two-stage region growing, *BAB* bio-inspired attention block, *MIB* multi-scale interactive block, *DSA* digital subtraction angiography.

We hereby present SE-RegUNet, an advanced architecture meticulously crafted for precise coronary segmentation. To improve image quality in noisy and low-contrast angiographic images, we have also introduced a novel two-step preprocessing technique that has been optimized for this purpose. The SE-RegUNet architecture innovatively incorporates squeeze-and-excitation (SE) blocks to improve feature learning, along with RegNet encoders to achieve a harmonious balance between accuracy and computational efficiency. To assess the practical applicability, we evaluated our model's efficiency and performed inference using publicly available angiogram datasets to verify its generalizability. Additionally, for a clearer understanding of how our architecture compares with the state-of-the-art models, we used uniform standards for assessment, complemented by external validation using publicly available datasets. Our goal is to advance medical diagnostic technologies by developing a coronary segmentation tool that combines high accuracy, broad applicability, and clinical efficiency, through improved model architecture and optimized image preprocessing.

## Materials and methods

### Study approval and data source

This study was approved by the Research Ethics Committee of China Medical University and Hospital in Taiwan (Document Number 1-REC1-92). All methods employed in this study were conducted strictly with relevant guidelines and regulations. We confirm that, based on the nature of our retrospective research and the anonymization of patient data, obtaining informed consent from subjects and/or their legal guardian(s) was deemed unnecessary by the Institutional Review Board (IRB) of China Medical University Hospital. The data source was coronary angiography performed at China Medical University Hospital (CMUH), a tertiary medical center in Taiwan, according to the clinical indications between 2021 and 2022. Of the 3793 consecutive angiographies selected, 1015 patients underwent PCI.

### Study dataset

As indicated in Table S2, previous research utilizing the U-Net framework has shown that a dataset of 600 angiogram videos can yield satisfactory training outcomes^12,14,17^. Typically, the first 6–10 videos from a cardiac catheterization are diagnostic angiographies. Therefore, we have decided to compile our dataset from 100 cardiac catheterization procedures, expecting to gather between 600 to 1000 angiography videos in total. In this study, we randomly collected 50 consecutive cases from two distinct groups in our database. The first group consisted of individuals with normal or mildly diseased coronary arteries, while the second group comprised individuals with severe coronary artery diseases who had undergone PCI. In total, we selected 100 cases to form our study cohort. Patients with a history of CABG were excluded from our selection. Only diagnostic angiograms were utilized for the development of our model. To establish ground truth for our models, three cardiologists independently selected a key frame image with optimal vessel opacification for each patient and created masks delineating coronary vasculature by consensus. We then used an open-access segmentation tool, MedSeg (https://www.medseg.ai/), to manually trace the vessels to produce ground truth masks. To develop a general segmentation model, all coronary arteries were labeled as one class. A total of 619 images of coronary angiography were labeled for this study.

### Model development

**Figure 1:**
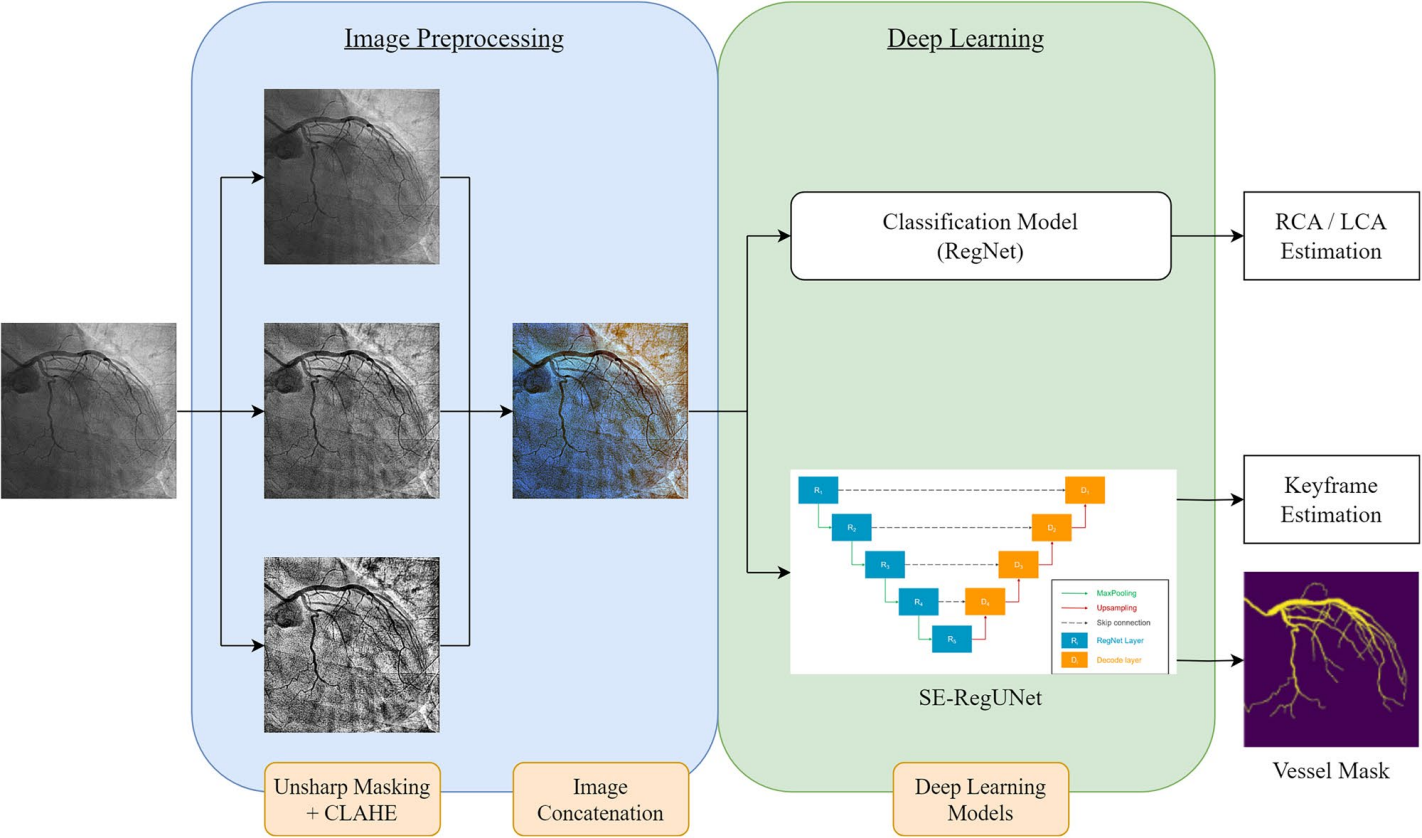
The flow diagram of the whole study design includes image preprocessing, a classification model, and a segmentation model. LCA, left coronary artery; RCA, right coronary artery.

We developed an automated analysis pipeline containing image preprocessing, classification, and segmentation models, as depicted in Fig. 1. As the first step, the raw angiography underwent image preprocessing to normalize contrast and enhance the vessel silhouette. Next, a classifier categorized each image as either a left or right coronary artery based on the main visible vessels. Once the left coronary artery (LCA) or right coronary artery (RCA) was determined, the corresponding segmentation model was applied to extract the coronary vasculature in that image.

#### Model training

We divided the dataset into training/validation and test sets to enable robust model development, optimization, and evaluation. The training/validation data, 80% of total images (510 angiography), was split into five equal folds for cross-validation. Each fold was iteratively held out for model validation, while the other four were used for training. This allowed for optimizing hyperparameters and assessing performance across different data combinations. After completing the cross-validation, we utilized the independent test set, comprising 20% of the total images (109 angiography images), which had been held out from the initial dataset division, to test the model. This final step aimed to provide an unbiased estimate of the model's performance on new, unseen data, further validating its generalizability and effectiveness in real-world scenarios. All experiments utilized high-performance computing platforms containing Intel® Xeon Platinum 8186 processors and Nvidia V100 Graphic Processing Units (GPUs) with 32 GB RAM to facilitate and ensure efficient training, cross-validation, and testing of the deep learning models on the angiography dataset.

#### Image pre-processing

**Figure 2:**
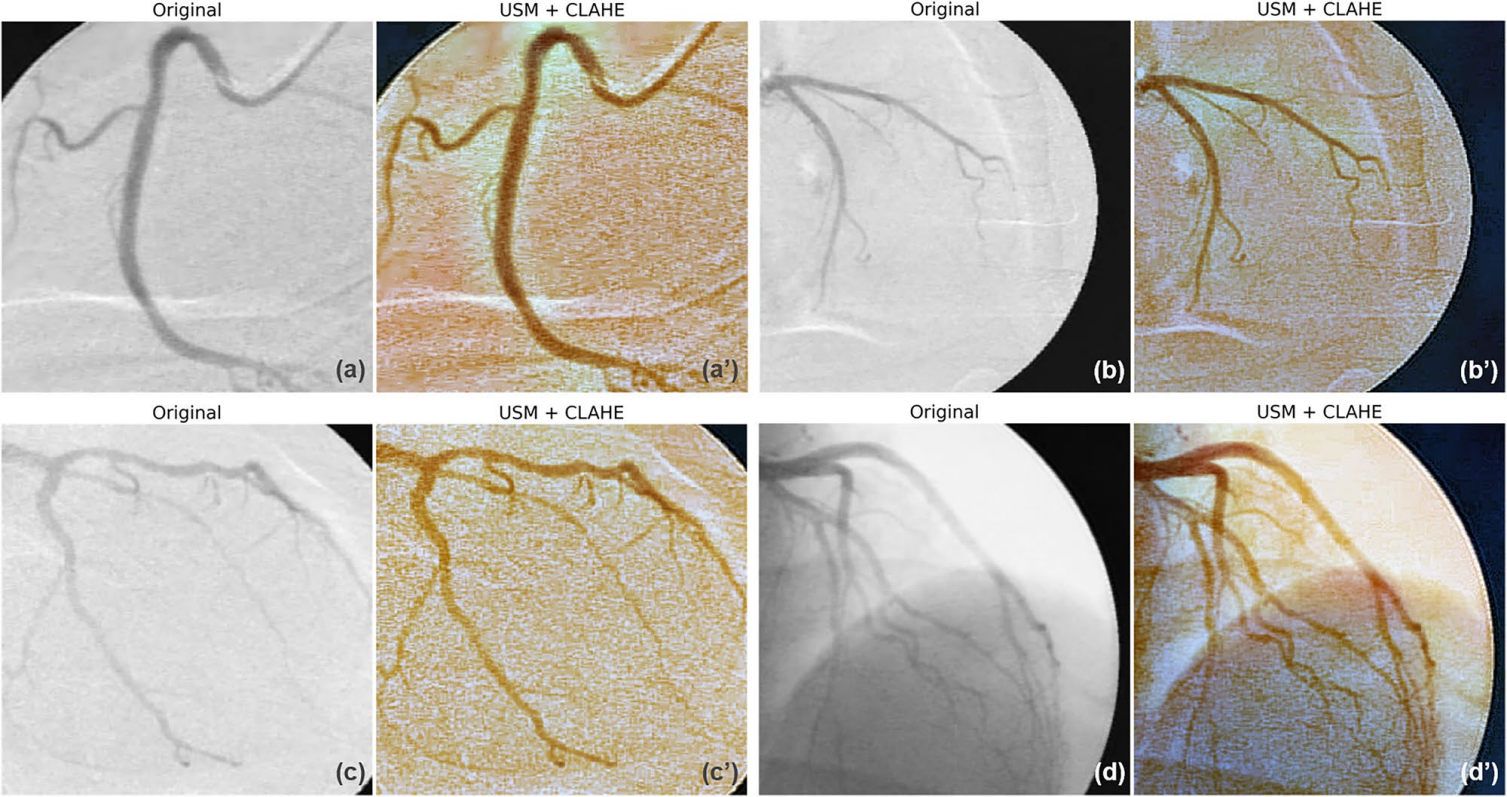
Comparison of coronary angiography images before (**a**–**d**) and after (**a′**–**d′**) image preprocessing steps. The application of USM + CLAHE visibly enhances the image quality, sharpening the edges and boundaries of the vessel for more precise analysis. USM, unsharp masking; CLAHE, contrast-limited adaptive histogram equalization.

We utilized an optimized preprocessing pipeline to normalize contrast and enhance coronary vessels on the raw angiography before input to the model. The pipeline combined two well-established techniques—unsharp masking (USM) and contrast-limited adaptive histogram equalization (CLAHE). USM, introduced by Malin in 1977, sharpens images by subtracting a Gaussian blurred version from the original image and merging the result. This enhances high-frequency details and edges^18^. CLAHE, developed by Zuiderveld^19^, adapts histogram equalization to local image tiles to boost contrast while limiting noise amplification. It was demonstrated that USM can significantly improve the perceived sharpness of an image by fusing with the first-order differential image and the second-order differential image, which makes the objects' edges appear more distinct and pronounced. CLAHE, on the other hand, is a variant of adaptive histogram equalization used to improve the contrast and visibility of details in digital images. CLAHE can help maintain detailed visibility and enhance the overall visual quality of the image while avoiding the noise and artifacts that can occur with standard histogram equalization. Both approaches were widely utilized in medical imaging for enhancing feature visibility^20–23^. Our pipeline applied USM followed by two CLAHE filters with tuned parameters. The resulting three images were merged into a 3-channel RGB image to be fed into the classification and segmentation models. Figure 2 shows this tailored preprocessing pipeline effectively extracted coronary vessels from the low-contrast angiography.

#### Coronary vessel segmentation model

**Figure 3:**
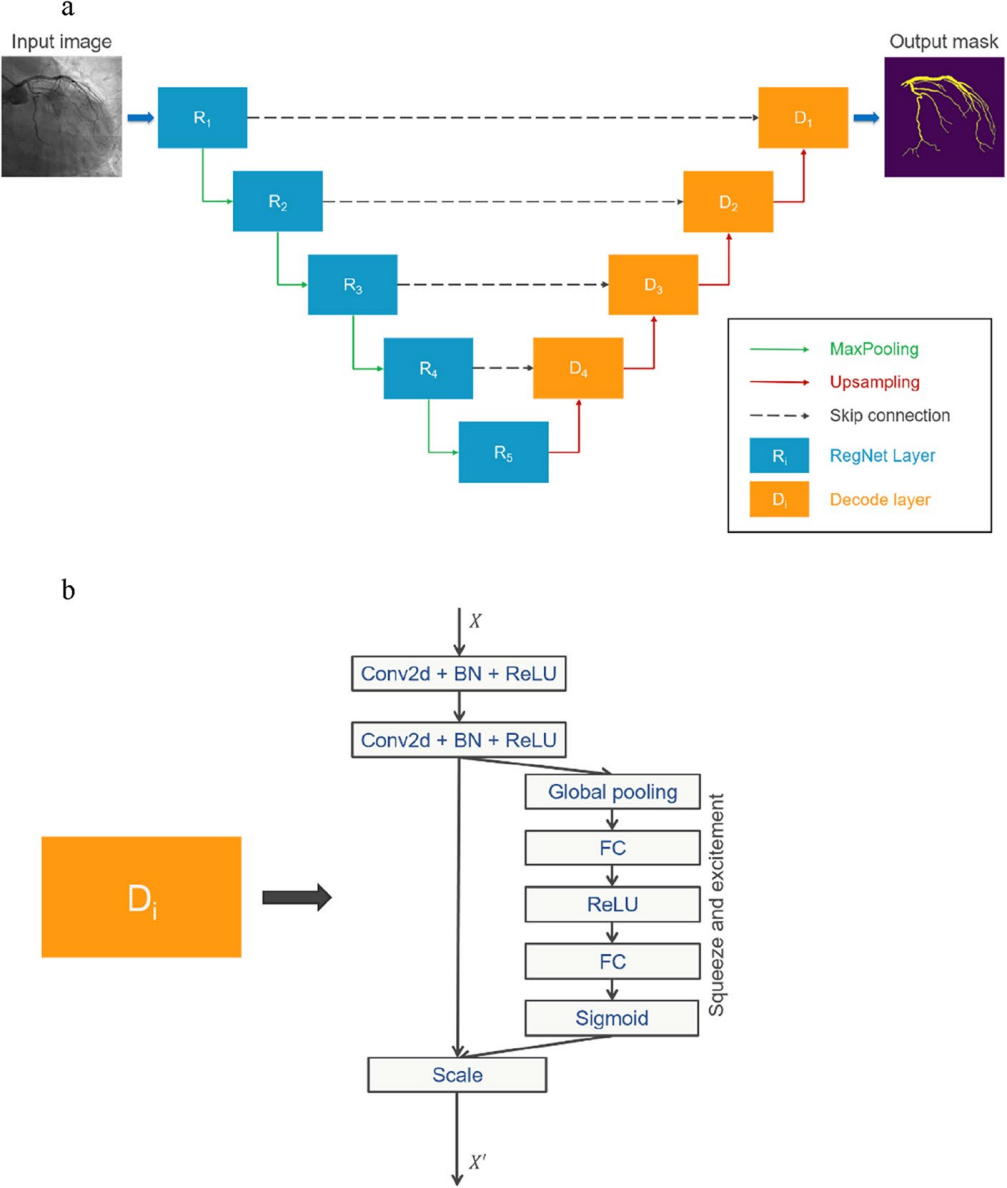
(**a**) The flow diagram of the SE-RegUNet model for coronary artery segmentation, comprising encoders and decoders linked by skip connections and incorporating self-attention modules to refine feature representations; (**b**) Illustration of the architectural composition of the decode layer. The decode layer consists of two 2D convolution layers with batch normalization and ReLU activation, forming the U-Net block. We enhance features by adding a squeeze and excitement block to the output.

This study introduced a novel SE-RegUNet model explicitly designed for efficient and accurate vessel segmentation. Ronneberger et al. introduced the U-Net architecture, which has become a popular segmentation model. U-net was built on the fully convolutional network, replacing pooling operators with upsampling operators in the decoder step. This increased the output resolution to match the input image, significantly improving biomedical image processing^24^. RegNet was later introduced by Facebook AI Research. This flexible convolutional network outperformed EfficientNet models with lower top-1 error rates and up to fivefold inferencing speed using GPUs while having similar floating-point operations per second (FLOPS)^25^. We replaced the encoder part of U-Net with this efficient and flexible RegNet backbone to enhance our model’s feature extraction capability. SE-RegUNet is a fusion of U-Net and RegNet, leveraging the strengths of both architectures. In addition, we introduced squeeze and excitement blocks in the decoder layer to adjust channel-wise feature responses by considering the interdependencies between channels in the model optimization process, focusing on relevant features, and reducing noise^26^. This unique design allows our model to adapt better to complex vessel structures. The structural diagram of the entire model and parts of the decoding layer are illustrated in Fig. 3. We developed two versions of models, SE-RegUNetz 4.0GF and SE-RegUNety 16GF models, for vascular segmentation.

The angiogram was meticulously annotated to differentiate between coronary artery vessels and non-vascular areas. A softmax function was added to the final layer of the segmentation model, which converts the raw scores into a probability distribution over the classes. The output values were between 0 and 1 and sum up to 1, making it easy to interpret as class probabilities. We adjusted the parameters of RegNet to match Meta® Research's GitHub repository “Classy Vision”^27^. During the training steps, we used 200 epochs with eight images per batch and a resolution of 512 × 512 pixels. Furthermore, to address the class imbalance, we employed a weighted focal loss (FL) during training to achieve better performance for the vessel segmentation task. This was formulated as shown:

1 $${\text{FL}} = { } - {\upalpha }\left( {1 - {\text{WCE}}} \right)^{\gamma } *\log \left( {{\text{WCE}}} \right)$$

where WCE means weighted cross entropy. The values for ${\upalpha }$ and ${\upgamma }$ were set at 0.8 and 2, and the weight of model loss in the vessel class was set at 20, respectively. We utilized Ranger21, a new optimizer developed by Wright et al. that combined AdamW with eight other components for model optimization^28^, with the learning rate set to 0.001.

For model comparisons, we used our data to train and benchmark other published state-of-the-art models for the same coronary arteries segmentation tasks, including AngioNet (angiographic processing network and DeepLabv3+ with Xception backbone)^12^, UNet3+^17^, UNet++ with EfficientNet-B5 backbone^14^, and Reg-SA-UNet++^29^.

### External validation

The UMAE T1-León Cardiology Department at the Mexican Social Security Institute has shared their DCA1 Dataset. This dataset includes 134 X-ray coronary angiography accurately labeled with segmentation ground truth by experienced cardiologists. Based on the dataset, Cervantes-Sanchez et al.^30^ were able to achieve an accuracy score of 0.9698 with a Dice coefficient of 0.6857 by employing a multilayer perceptron network and several preprocessing methods including Gaussian matched filters and Gabor filters. We used this database as our external validation dataset to compare our model performance to other state-of-the-art models. All the comparison models tested in this study were trained from scratch to ensure that the pre-trained weight with optimized parameters did not affect the model performance. To prevent overfitting in a limited dataset, the training process for this section was capped at 100 epochs.

#### Evaluation metrics

We employed several key metrics in our model evaluation, including sensitivity (recall) (S1), specificity (S2), accuracy (S3), and precision (S4)^31^. When evaluating the segmentation model, we utilized the Sørensen-Dice coefficient, also known as the Dice score, a commonly used statistical tool, computed using a formula:

2 $${\text{Dice}} = { }\frac{{2{*}\left| {X \cap Y} \right|}}{\left| X \right| + \left| Y \right|}$$

This formula compares the ground truth (X) against the prediction generated by the segmentation model (Y), which is presented as a probability map ranging from 0 to 1. LCA and RCA classification performance was measured using Area Under the Receiver Operating Characteristic Curve (AUC)^32^ and accuracy (S3) as metrics.

## Results

Table 2 displays the baseline characteristics of the 100 patients employed in this study. The average age of the cohort is 65.2 years, with the majority being male (69%). The data also reveals a high prevalence of cardiovascular risk factors, such as diabetes (38%), hypertension (63%), and chronic kidney disease (23%).

**Table 2:** Descriptive characteristics of the dataset used for model development.

	N ± SD or N (%)
Age (y/o)	65.2 ± 13.5
Sex	Male: 69 (69) Female: 31 (31)
BMI	25.2 ± 4.2
Diabetes mellitus	38 (38)
Hypertension	63 (63)
Chronic kidney disease	23 (23)
Acute myocardial infarction	5 (5)
LVEF (%)	54.2 ± 11.7

BMI, body mass index; LVEF, left ventricular ejection fraction.

Table S1 compares different models' computational demands and complexities based on their number of parameters, FLOPs, model size and training memory used. The SE-RegUNety 16GF model showed significantly more parameters (196.3 million) than the other models, while AngioNet was found to be the most efficient with 11.0G FLOPs. Among the different versions of U-Net models, the SE-RegUNetz 4GF exhibited the most well-rounded performance with moderate parameters (30.6 million) and FLOPs (28.0). Compared to similar model architectures, the SE-RegUNetz 4GF demands less memory than most of these models, hinting at its suitability for training on a smaller GPU. Based on the complexity and inference speed of the model employed, we selected the RegNetz 4GF-based U-Net model, denoted as “SE-RegUNet 4GF,” as our primary model from the two RegNet model structures we introduced.

LCA and RCA classification serves as the foundation for automatic coronary angiography assessment. In this task, 80% of the images were used for training and validation, with the remaining 20% reserved for testing. This classification model achieved an AUC score of 1.000 and 99.08% accuracy in determining whether the coronary artery is LCA or RCA.

**Figure 4:**
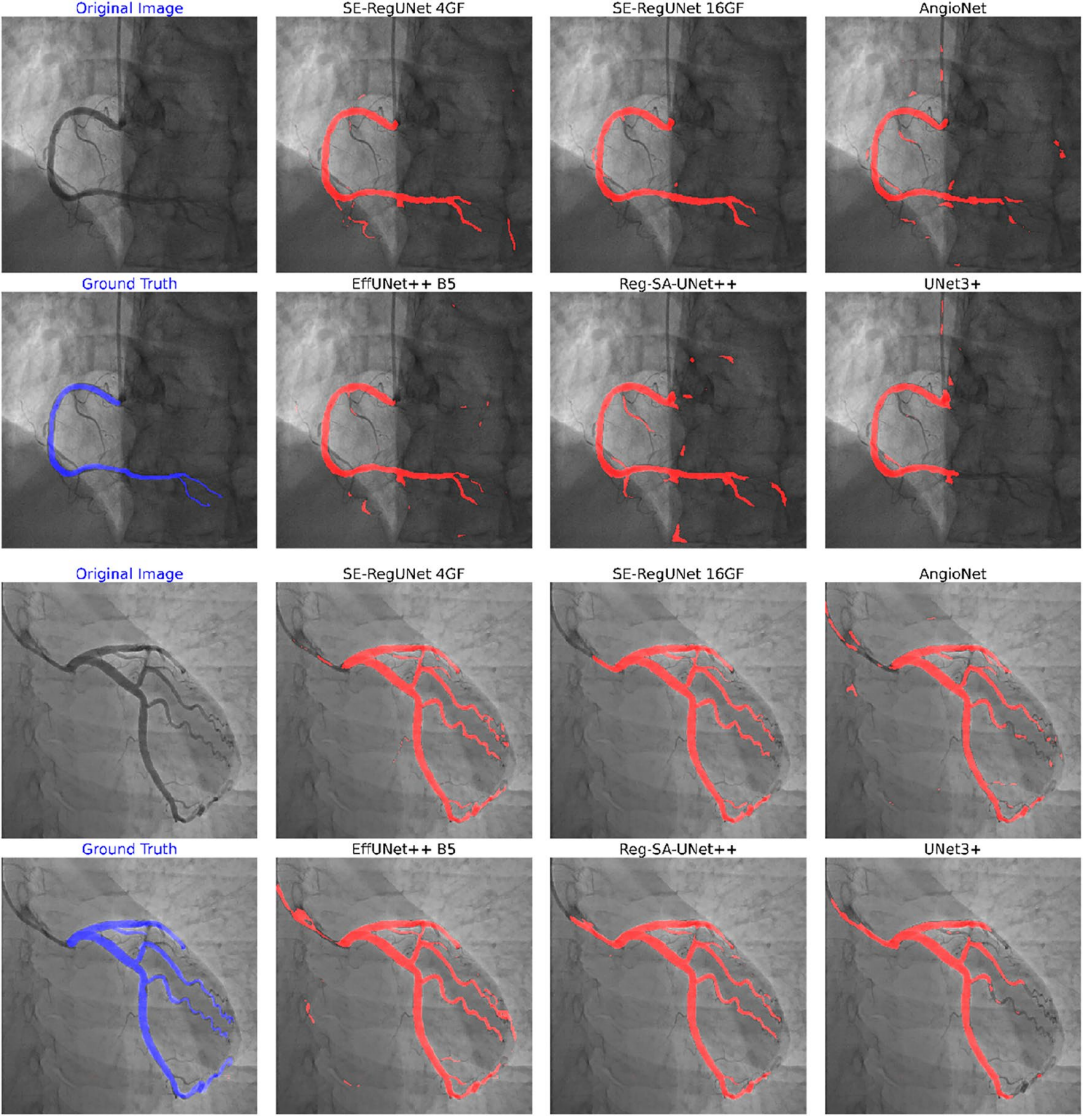
Comparison of ground truth (blue mask) and the segmentation results of each selected model (red mask) for the right coronary artery (top portion) and the left coronary artery (bottom portion).

Table 3 summarizes the segmentation performance and efficiency of the different models tested. The SE-RegUNet 4GF model achieved the best overall Dice score (0.7217) and accuracy (0.9721) while maintaining a high efficiency with a frame per second (FPS) of 41.6. In contrast, SE-RegUNet 16GF had top specificity (0.9907) but lower FPS due to its large model architecture. Figure 4 visually compares the ground truth masks (in blue) and segmentation results (in red) from each model for both left and right coronary arteries. The SE-RegUNet 4GF model showed great qualitative results overall.

**Table 3:** Evaluation of model segmentation performance and efficiency.

Model (Backbone)	Dice score (SD)	Accuracy (SD)	Sensitivity (SD)	Specificity (SD)	Precision (SD)	FPS* (SD)
AngioNet (Xception)	0.6273 (0.0946)	0.9560 (0.0110)	0.7518 (0.0936)	0.9684 (0.0109)	0.5615 (0.1345)	60.8259 (2.4769)
UNet3+	0.6353 (0.1153)	0.9606 (0.0131)	0.6996 (0.1456)	0.9759 (0.0125)	0.6217 (0.1418)	13.1622 (0.2059)
UNet++ (EfficientNet-B5)	0.7090 (0.0770)	0.9707 (0.0103)	0.7116 (0.1134)	0.9863 (0.0069)	0.7370 (0.1208)	24.5961 (1.3603)
Reg-SA-UNet++ (RegNetz 4GF)	0.6822 (0.0992)	0.9682 (0.0121)	0.6878 (0.1360)	0.9849 (0.0103)	0.7170 (0.1307)	28.8096 (2.0621)
SE-RegUNety 16GF (RegNety 16GF)^+^	0.7034 (0.1006)	0.9722 (0.0112)	0.6585 (0.1383)	0.9907 (0.0049)	0.7905 (0.1075)	25.0908 (1.0991)
SE-RegUNetz 4GF (RegNetz 4GF)^+^	0.7217 (0.0773)	0.9721 (0.0096)	0.7240 (0.1137)	0.9869 (0.0062)	0.7477 (0.1141)	41.6606 (2.6358)

*FPS* frame per second, *SD* standard deviation.

*FPS data was collected using a single NVIDIA V100 GPU.

+The models that were developed in our study.

To demonstrate the real-world clinical application of our developed models, we implemented an interactive web-based tool using the HuggingFace platform (https://huggingface.co/spaces/KurtLin/CoronaryAngioSegment) (Fig. S2). This prototype application enabled users to upload a coronary angiographic image and obtain vessel segmentations from our best-performed model, potentially assisting physicians in artery visualization and analysis during their clinical assessment. However, extensive clinical validation is still required before clinical implementation. On average, our SE-RegUNet 4GF model took around 2 s to generate masks for an image with a 512 × 512 pixels resolution. This was achieved using only two vCPUs and 16 GB of RAM.

We further evaluated another integrating the angiographic processing network (APN) from AngioNet as an additional preprocessing step. APN applies adaptive filtering before segmentation, unlike our static pipeline of USM and CLAHE. Table 4 shows that adding APN before our model improved sensitivity, resulting in fewer false negatives. However, this also led to a lower overall Dice score than USM + CLAHE.

**Table 4:** Comparison of segmentation performance with different image preprocessing techniques for coronary angiography.

Model (Backbone)	Preprocessing	Dice score (SD)	Accuracy (SD)	Sensitivity (SD)	Specificity (SD)	Precision (SD)
SE-RegUNet (RegNetz 4GF)	APN	0.6489 (0.1067)	0.9640 (0.0098)	0.8493 (0.0691)	0.9698 (0.0091)	0.5431 (0.1366)
	USM + CLAHE	0.7217 (0.0773)	0.9721 (0.0096)	0.7240 (0.1137)	0.9869 (0.0062)	0.7477 (0.1141)
	USM + CLAHE + APN	0.7297 (0.0793)	0.9718 (0.0096)	0.7597 (0.0969)	0.9846 (0.0074)	0.7267 (0.1254)

*APN* angiographic processing network, *USM* unsharp masking, *CLAHE* contrast-limited adaptive histogram equalization, *SD* standard deviation.

When validated on the independent DCA1 dataset, SE-RegUNet 4GF again achieved the highest Dice coefficient (0.76) and sensitivity (0.78) among all models tested, as shown in Table 5. However, the AngioNet model showed lower performance on external data, with the lowest Dice score (0.66) and sensitivity (0.61). UNet3+ and Reg-SA-Unet++ achieved similar overall Dice scores of around 0.75, while UNet++ scored 0.74. All tested models showed a minor decrease in Dice score and sensitivity compared to internal cross-validation, as often observed when validating the new dataset.

**Table 5:** Comparison of segmentation performance of models validated on the DCA1 dataset.

Model (Backbone)	Dice score	Accuracy	Sensitivity	Specificity	Precision
AngioNet (Xception)	0.6624	0.9676	0.6121	0.9891	0.7751
UNet3+	0.7187	0.9712	0.7169	0.9862	0.7728
UNet++ (EfficientNet-B5)	0.7420	0.9723	0.7484	0.9851	0.7523
Reg-SA-UNet++ (RegNetz 4GF)	0.7595	0.9731	0.7764	0.9850	0.7537
SE-RegUNet 16GF^*^ (RegNety 16GF)	0.7557	0.9730	0.7718	0.9853	0.7582
SE-RegUNet 4GF* (RegNetz 4GF)	0.7621	0.9730	0.7821	0.9849	0.7537

*The models that were developed in our study.

## Discussion

This study clearly demonstrated a novel deep-learning approach of SE-RegUNet architecture for the automatic segmentation of coronary arteries in angiography, which achieved top performance with a Dice score of 0.7217 and an accuracy of 0.9721 on the test dataset. A key contribution of this study was integrating RegNet encoders and squeeze-and-excitation blocks to boost segmentation accuracy while maintaining high efficiency. Another important novelty was our optimized image preprocessing pipeline combining USM and CLAHE to extract and enhance vessel structures from noisy, low-contrast angiography.

A deep learning model's total number of parameters could affect its complexity, capacity, and learning ability. In general, models with more parameters have higher representational power as they can capture the hidden patterns and relationships in the dataset. However, more parameters could cause the model to overfit easily and have a slower infer speed. In our dataset, the SE-RegUNet 16GF model achieved the highest Dice score, accuracy, specificity, and precision in the test dataset. However, the model had the second lowest infer speed due to its complex model structure. AngioNet, which combined APN with Xception-backboned DeepLabv3+, can analyze 60 images per second but has the lowest Dice score and sensitivity. Overall, SE-RegUNet 4GF was the most balanced model in our study, with the second-highest Dice score, accuracy, sensitivity, specificity, and precision evaluated in the testing set. External validation on the DCA1 Dataset also showed similar results, in which SE-RegUNet 4GF scored the highest Dice score and sensitivity. In this case, SE-RegUNet 16GF showed lower performance than SE-RegUNet 4GF, possibly due to the smaller external sample size, which is only one-fifth of the CMUH dataset. The model with more parameters will easily overfit with a small dataset since the model only learns the specific patterns in the training data and thus may not perform well in the external validation dataset. For potential real-world applications in the cath lab, we also try to add a function to detect the best keyframe for evaluating the segmentation model of coronary arteries. After testing in the CMUH dataset, the keyframe determination task had a mean absolute error (MAE) of 6.5 frames (0.433 s), as shown in Fig. S3, comparable to the results reported by other researchers^16,33^. We will include this step in our pipeline as part of our upcoming functionality.

To address the challenges encountered in previous studies, where models demonstrated diminished performance in scenarios of poor image quality or when the contrast between vessels and background was low^13,14^, we incorporated two image preprocessing steps in addition to customizing our model. This research conducted a comparative analysis between our approach and the Adaptive Preprocessing Normalization (APN), which has been reported to exhibit commendable performance^12^. The results indicated that the APN approach had a higher sensitivity compared to our solution, which involved two steps of USM and CLAHE. However, our method achieved a better overall Dice score. The combination of APN and USM + CLAHE yielded only minor improvements in Dice and sensitivity, while significantly reducing efficiency. Using adaptive techniques like APN can improve specific metrics, however, our optimized static preprocessing pipeline still provides competitive coronary vessel segmentation performance with high efficiency.

The accuracy of the automatic vessel segmentation task in coronary angiography has been extensively studied. For example, Iyer et al.^18^ introduced AngioNet, which combined a self-designed APN preprocessing method with Deeplabv3+ and improved Dice scores from 0.812 to 0.864. Meng et al.^17^ achieved a Dice score of 0.8942 using U-Net 3+ with full-scaled skip connections and deep supervisions. Menezes et al.^34^ used U-Net++ with EfficientNet B5 backbone, obtaining a Dice score of 0.8904 with the original dataset, and after data augmentation and fine-tuning, the score increased to 0.9134. However, different evaluating metrics and vessel mask principles were used in these studies, making it difficult to compare the achieved results among varied models. Moreover, some of the research relies on small training data sets and does not provide performance results on external data sets^16,35,36^, raising concerns about the models' ability to generalize. Therefore, in this research, we re-trained all the comparison models with our CMUH study Dataset and validated them on an independent DCA1 Dataset. The proposed SE-RegUNet 4GF achieved the highest Dice score and accuracy on the CMUH dataset. When validated on independent DCA1 data, SE-RegUNet 4GF again showed the highest Dice and sensitivity and high accuracy. Our model's ability to maintain consistent segmentation performance, regardless of the data source, indicated its overall generalizability. While all the tested models showed slightly reduced performance metrics when validated on external data, our tailored approach still maintained good accuracy.

## Limitations

Our study has inherent challenges in using deep-learning models to segment coronary arteries from angiography. First, when filled with contrast medium, the catheters connected to the left main (LM) artery or RCA have characteristics similar to those of the adjacent coronary vessels, making it difficult for the segmentation model to classify them accurately. Even when the catheter mask was removed during training steps, the model still misclassified some parts of the catheter as a vessel, as shown in Fig. S4. Identifying the ostial section of the coronary arteries (such as LM and ostial RCA) from the catheter can be challenging. In this case, categorizing catheters as a distinct class during model training could help differentiate them from vessels. Second, we chose to exclude patients who have undergone CABG surgery. This is due to the complexity of the vasculatures and the need to pan the exam table to cover the entire course of the graft vessels. This could potentially limit the generalizability of the models. However, this could be addressed by studying videos or selecting multiple key images in the future studies. Third, if the ribs overlay directly on the vessel, it can cause a contrast difference, which may result in the model's prediction showing a fractured mask near the border. Finally, despite cardiologists agreeing on labeling methods, some minor discrepancies remain in their labeling details. For example, some cardiologists labeled all blood vessels, while others only labeled the clinically significant arteries. To address this, prior discussions on labeling methods should have taken place to minimize these variations. Another limitation of this study was that the models were developed solely based on a dataset gathered from a solitary medical facility. The data set comprised only 100 patients and was collected using a single X-ray machine within a year. As a result, the findings may not apply to other hospital settings. However, we did use an external dataset from a different country for validation. It helped to ensure that our model's performance was still acceptable in other hospital settings. Nevertheless, we plan to conduct further external validations before considering them for real-world clinical use.

## Future works

Although the current model for vessel segmentation exhibits high accuracy and efficiency, additional features are necessary to develop a comprehensive automated assistant for coronary angiography in clinical settings. The next step should be to expand the platform to classify stenosis severity and make it an integrated cardiovascular risk assessment and treatment recommendation tool. It is vital to validate the system across diverse patient populations in different hospital settings and imaging equipment to evaluate its real-world performance. The design must be seamlessly integrated into routine clinical workflows and meet regulatory requirements before it can be widely adopted as the new standard of care.

## Conclusion

The SE-RegUNet 4GF model shows excellent potential in helping physicians efficiently and accurately detect coronary artery disease by segmenting coronary angiography. However, further refinements to the model optimization with extensive clinical validations are needed before it can be integrated into regular clinical practices.

## Supplementary Information

Supplementary Information.

## Data Availability

The dataset used in this study, obtained from China Medical University Hospital, is currently unavailable due to privacy concerns. However, we utilized the DCA1 dataset for external validation, which can be accessed through the link provided by the author at http://personal.cimat.mx:8181/~ivan.cruz/DB_Angiograms.html. If interested, the code can be made available by the corresponding author upon request.
