# Supplementary material for: Optimizing ensemble U-Net architectures for robust coronary vessel segmentation in angiographic images
Source: Sci Rep. 2024 Mar 19;14:6640. doi: 10.1038/s41598-024-57198-5 (PMC10951254; doi:10.1038/s41598-024-57198-5)
Supplement: Supplementary file 1 — Supplementary Information. [file 41598_2024_57198_MOESM1_ESM.docx]

**Optimizing Ensemble U-Net Architectures for Robust Coronary Vessel Segmentation in Angiographic Images**

## Equations for the accuracy metrics:

$sensitivity= \frac{TP}{TP+FN}$ (S1)

$specificity= \frac{TN}{TN+FP}$ (S2)

$accuracy= \frac{TP+TN}{TP+FP+FN+TN}$ (S3)

$precision= \frac{TP}{TP+FP}$ (S4)

Counting pixel-wised, true positive (TP) is the number of true positives, false positive (FP) is the number of false positives, true negative (TN) is the number of true positives, and false negative (FN) is the number of false negatives.

## Supplement tables and figures

### Table S1. Comparison of Model Complexity and Computational Cost for Coronary Vessel Segmentation Architectures

| Model (Backbone) | # of Parameters (M) | FLOPs (G) | Model Size (MB) | Training Memory Used* (GB) |
| --- | --- | --- | --- | --- |
| AngioNet (Xception) | 41.2 | 11.0 | 141.7 | 8.00 |
| UNet3+ | 27.0 | 799.4 | 103.0 | 46.67 |
| UNet++ (EfficientNet-B5) | 2.4 | 15.5 | 113.7 | 18.21 |
| Reg-SA-UNet++ (RegNetz 4GF) | 30.1 | 40.2 | 121.4 | 22.00 |
| SE-RegUNet 16GF (RegNety 16GF)^+^ | 196.3 | 305.4 | 773.6 | 13.14 |
| SE-RegUNet 4GF (RegNetz 4GF)^+^ | 30.6 | 28.0 | 118.9 | 12.30 |

FLOPs, floating-point operations per second; M: million; G, giga: 10^9^.

+ The models that were proposed in our study.

*Memory usage was measured for RGB images with 512x512 resolution and a batch size 8.

### Table S2. Number of Coronary Angiography Frames Used for Each Segmentation Task

| Reference | Model | Amount of Dataset |
| --- | --- | --- |
| Iyer et al., 2021^12^ | AngioNet | 462 frames |
| Meng et al., 2023^17^ | U-Net 3+ | 616 frames |
| Menezes et al., 2022^14^ | U-Net++ | 416 frames |
| **Our research** | ***SE-RegUNet*** | ***619 frames*** |

### Figure S1


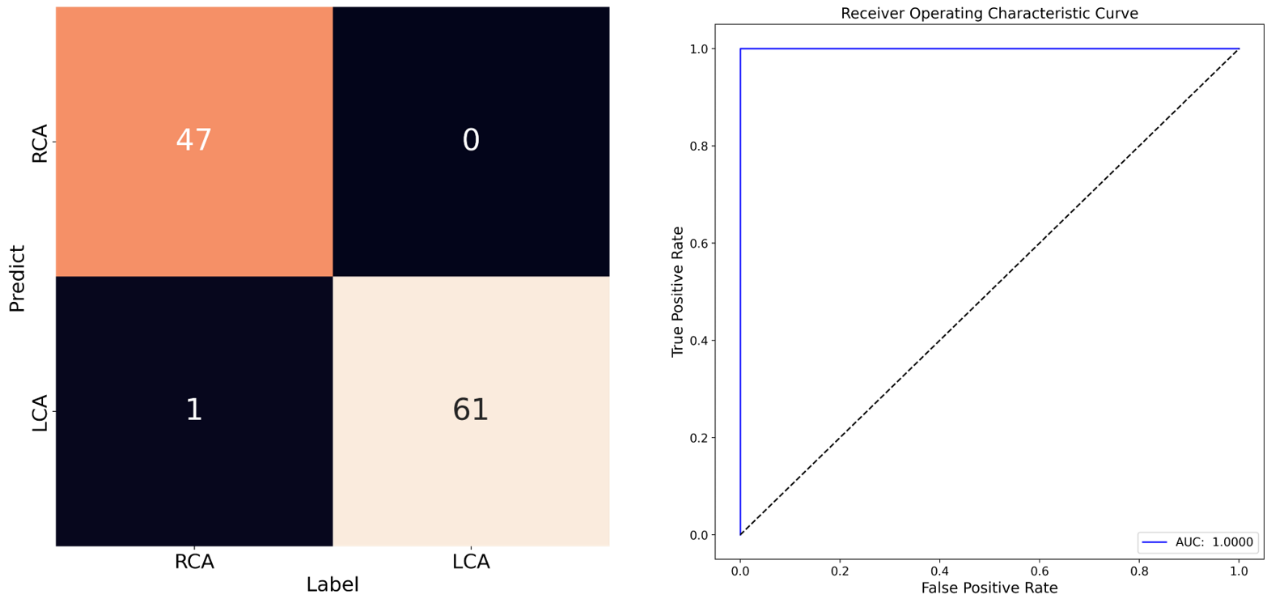


Figure S1. Confusion Matrix and ROC Curve of the LCA/RCA Classification Model. LCA, left coronary artery; RCA, right coronary artery.

### Figure S2


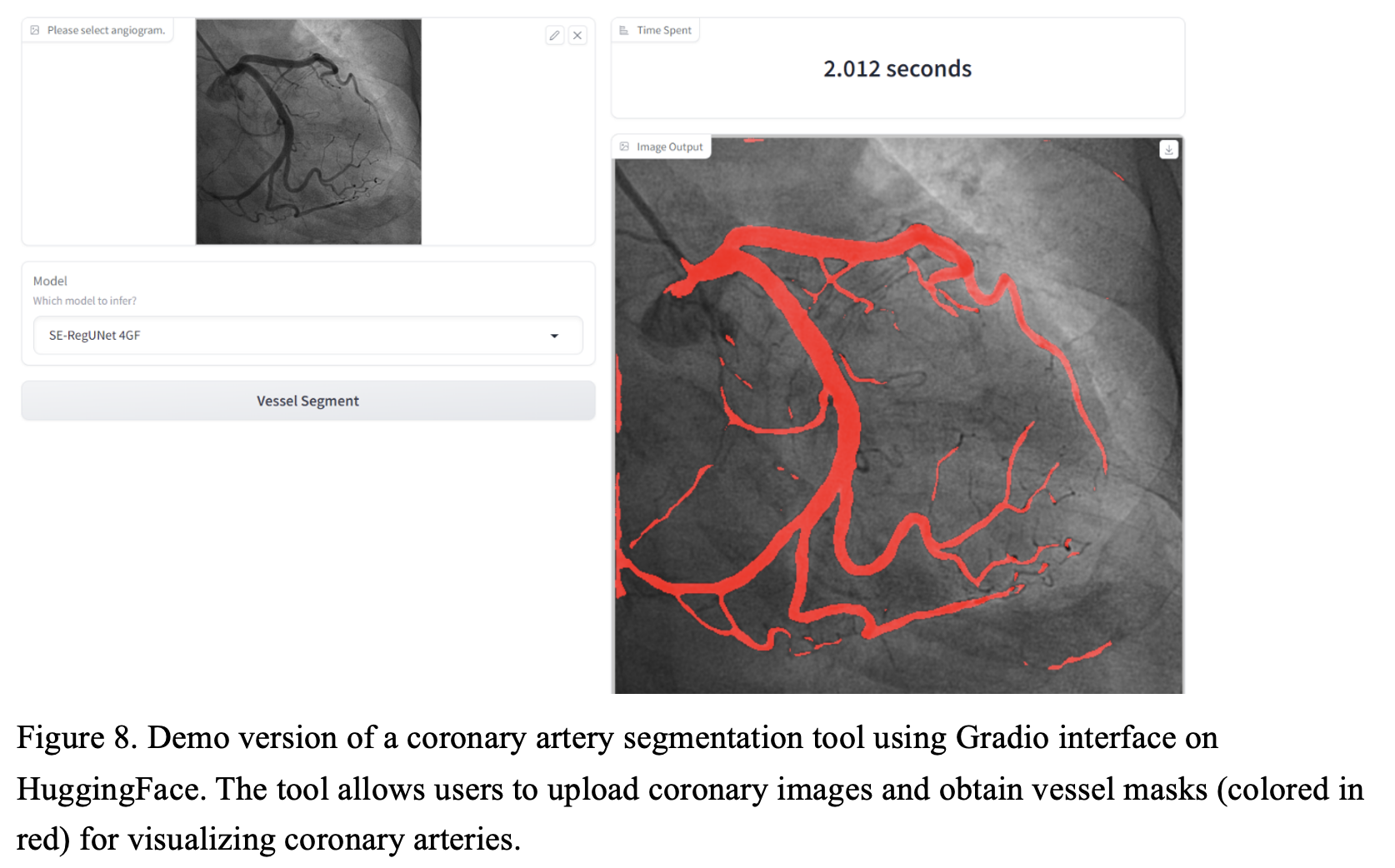


Figure S2. Demo version of a coronary artery segmentation tool using Gradio interface hosted on HuggingFace. The tool allows users to upload coronary images and obtain vessel masks (colored in red) for visualizing coronary arteries.

### Figure S3


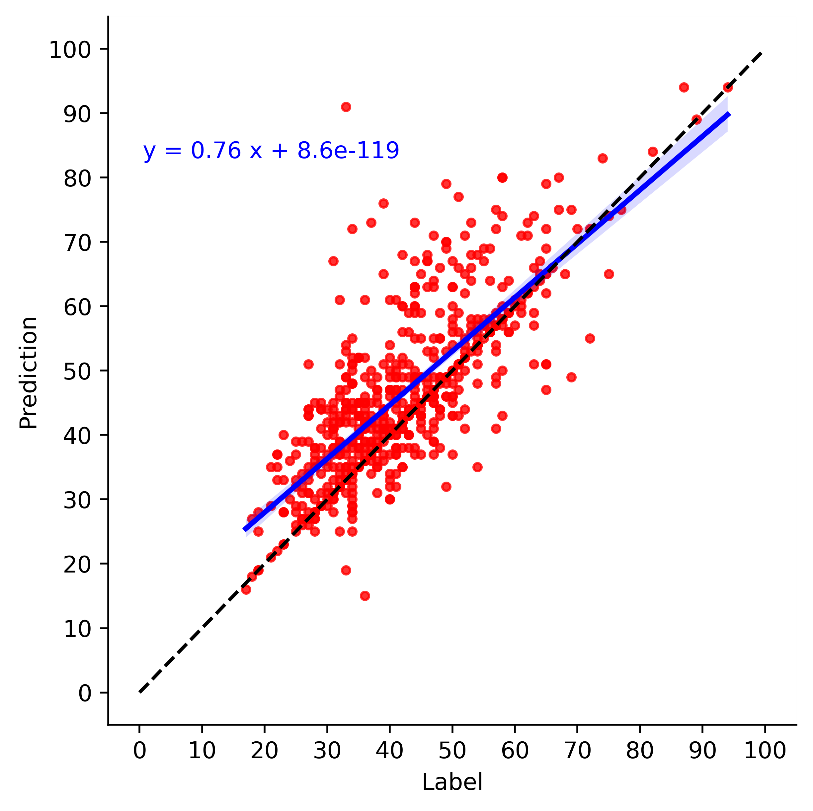


Figure 3. Scatter Plot Illustrating the Correlation between Predictions and Keyframe Indices from the Labeled Dataset

### Figure S4


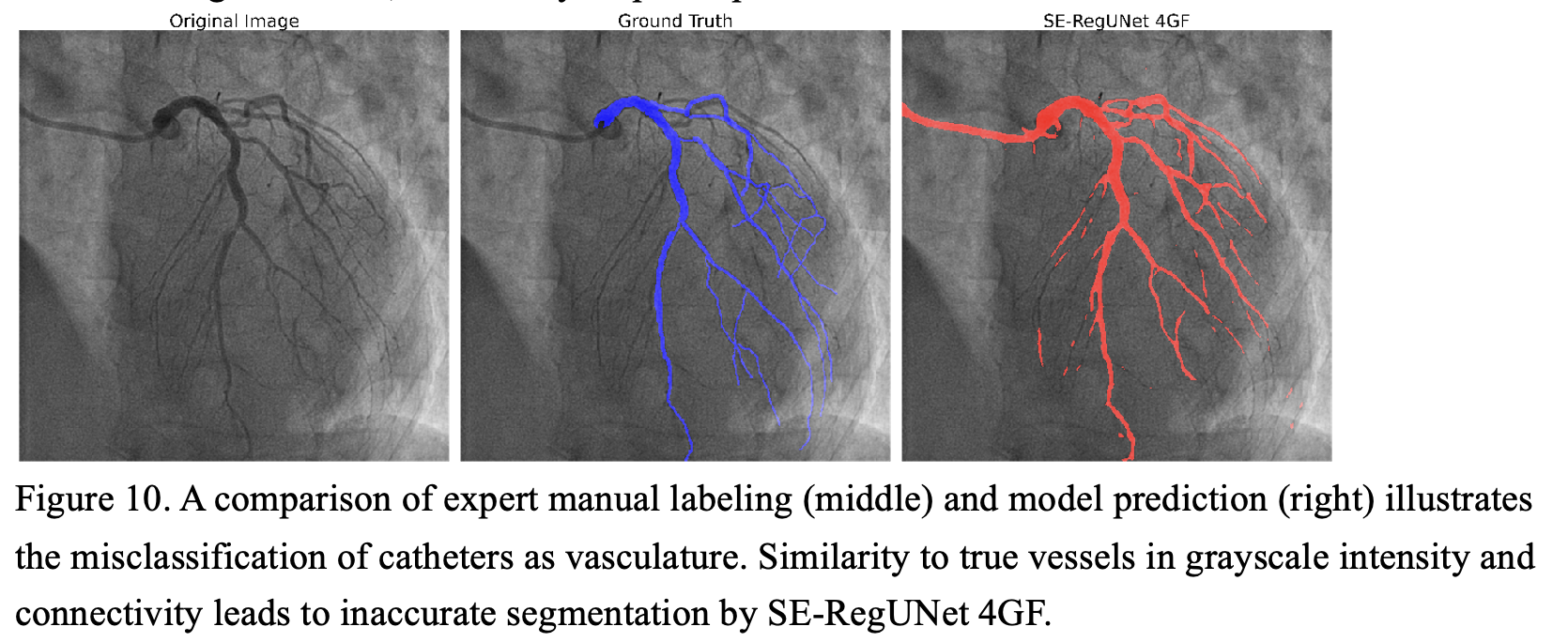


Figure S4. A comparison of expert manual labeling (middle) and model prediction (right) illustrates the misclassification of catheters as vasculature. Similarity to true vessels in grayscale intensity and connectivity leads to inaccurate segmentation by SE-RegUNet 4GF.
